# Rethinking Catalysis in Three-Dimensional Topological Materials Through Surface Oxidation

**DOI:** 10.3390/nano16171092

**Published:** 2026-09-01

**Authors:** Danil Boukhvalov, Kazybek Aimaganbetov, Vladimir Yu. Osipov, Antonio Politano

**Affiliations:** 1 Institute of Materials Physics and Chemistry, College of Science, Nanjing Forestry University, Nanjing 210037, China; 2 Institute of Physics and Technology, Satbayev University, Almaty 050032, Kazakhstan; 3 International Research and Educational Center for Physics of Nanostructures, ITMO University, 197101 St. Petersburg, Russia; 4 Department of Physical and Chemical Sciences, University of L’Aquila, Via Vetoio, 67100 L’Aquila, Italy

**Keywords:** topological materials, oxidation, DFT

## Abstract

The catalytic activity of topological materials is often attributed to the nontrivial electronic states of pristine surfaces. However, this interpretation does not explain why chemically and structurally distinct topological compounds frequently exhibit similar catalytic performance despite substantial differences in their band topology. A key limitation of this view is the neglect of surface reconstruction, particularly oxidation under ambient conditions, which can significantly modify both the electronic structure and the nature of active catalytic sites. Here, density functional theory calculations were performed to investigate the energetics of surface oxidation and the hydrogen evolution reaction in representative three-dimensional topological materials, including VAl_3_, PtGa, PtAl, NbAs, TaAs, and NbP. Surface formation generates highly reactive sites that promote oxygen dissociation and spontaneous oxidation. Although oxidation substantially reconstructs the electronic structure near the Fermi level, favorable hydrogen adsorption energetics are preserved for most compounds and, in some cases, further improved at high hydrogen coverage. These findings identify surface oxidation as a common chemical response across topological materials and provide a unified explanation for their experimentally observed catalytic activity. The results highlight that catalytic performance arises from the combined effects of the parent electronic structure and the chemistry of the oxidized surface, rather than from pristine topological surface states alone.

## 1. Introduction

The development of effective catalysts increasingly depends on controlling composition, morphology, active-site density, and electronic states that influence adsorption, charge transfer, and bond activation. Within this framework, topological materials [1] have attracted increasing attention as catalytic platforms due to their nontrivial bulk and surface electronic structures, which can provide unique pathways for interfacial charge transfer and chemical reactions [2]. Most studies of topological catalysis therefore seek correlations between catalytic performance and specific electronic-structure characteristics, including strong spin–orbit coupling, Weyl-semimetal states, and surface Fermi arcs [3,4,5]. From a structural perspective, topological materials can be broadly divided into layered and three-dimensional non-layered systems. Layered materials, represented mainly by transition-metal dichalcogenides, expose surfaces through the cleavage of weak van der Waals interactions [6]. In contrast, surface formation in three-dimensional materials, such as PtGa, requires breaking metallic or covalent bonds, thereby producing undercoordinated surface atoms and chemically reactive dangling bonds. These surfaces can simultaneously host nontrivial electronic states, including Fermi arcs [7]. Therefore, their electronic structure and catalytic response are strongly dependent on the exposed crystallographic facet [8].

Despite the considerable attention devoted to topological electronic states, the chemical stability of the surfaces of topological materials and their reconstruction upon interaction with the environment have been systematically overlooked. This omission is particularly critical for three-dimensional topological materials, whose preparation or cleavage necessarily disrupts metallic or covalent bonding and can promote rapid reactions with atmospheric species. For example, in the Nb(Ta)As(P) family, theoretical studies have associated catalytic activity with surface Fermi arcs, whereas the corresponding experimental characterization has often been limited to X-ray diffraction and spectroscopy of nominally as-cleaved surfaces [9,10]. Nevertheless, X-ray photoelectron spectroscopy has revealed oxide-related components, while density functional theory calculations have predicted favorable oxygen adsorption and dissociation on NbAs surfaces [11]. Similar evidence of surface oxidation has been reported for other non-layered topological materials, including Ru_3_Sn_7_ [12], OsCoTe_2_ [13], SnTe [14], noble-metal compounds, nickel stanenes [15,16], NiV [17], and CoS [18]. Energy-dispersive X-ray spectroscopy has likewise revealed substantial oxygen contents in VAl_3_ [19], PtAl, and PdGa [20], although their catalytic properties have generally been interpreted using models based on pristine, oxygen-free surfaces [19,20]. These observations point to a common behavior across chemically and structurally distinct systems: the surfaces relevant to catalysis are frequently reconstructed or oxidized before catalytic measurements are performed. Therefore, surface oxidation must be considered when assigning catalytic activity to topology-derived electronic states.

Resolving this fundamental inconsistency is essential because any interpretation based exclusively on pristine surfaces risks attributing the observed catalytic activity to topological electronic states that are no longer present in the actual, chemically transformed catalyst.

Here, we address the chemical stability, oxidation energetics, and hydrogen-evolution properties of representative three-dimensional topological materials, including VAl_3_, PtGa, PtAl, NbAs, TaAs, and NbP.

## 2. Materials and Methods

The atomic structures, surface energetics, and reaction energetics were investigated within density functional theory using the Quantum ESPRESSO 6.7 package [21]. Exchange–correlation effects were described within the generalized-gradient approximation using the Perdew–Burke–Ernzerhof functional [22], including van der Waals corrections. Ultrasoft pseudopotentials were employed for all elements [23], with kinetic-energy cutoffs of 35 and 400 Ry for the wave functions and charge density, respectively. This computational code, functionals, and pseudopotentials have been used previously in our works on the oxidation and catalytic properties of similar materials [11,15,16]. Systematic comparison of PBE pseudopotentials with the PBEsol functional implemented in different codes shows deviations in materials formation energies of <0.1 eV/atom, which are much smaller than the magnitudes of the energies reported in Table 1 [24,25]. Regarding surface energies, both types of pseudopotentials underestimate their magnitudes [24,25]. Thus, the use of the PBE functional results in about a 10% larger underestimation of surface stability. Therefore, the tendencies to oxidation discussed in this work are surely not associated with peculiarities of pseudopotentials. For the description of non-covalent bonds, +vdW corrections are obviously essential [25].

Reaction enthalpies were evaluated as the difference between the total energies of the products and the reactants; negative values therefore indicate exothermic processes.

The physisorption enthalpy of an isolated molecular species was calculated as

ΔH_phys_ = E_host+mol_ − (E_host_ + E_mol_), (1)

where E_host+mol_ is the total energy of the adsorbate–surface system, E_host_ is the energy of the pristine surface, and E_mol_ is the energy of the isolated molecule calculated in the same simulation cell. For molecular physisorption, the corresponding Gibbs free-energy change was estimated as

ΔG = ΔH − TΔS,(2)

where T is the temperature, and ΔS is the entropy change associated with adsorption. Within the approximation adopted here, the entropy loss was estimated by considering the gas-to-liquid transition according to

ΔS = ΔH_vap_/T, (3)

where ΔH_vap_ is the experimental enthalpy of vaporization.

The oxygen-dissociation (chemisorption) energy was obtained from the difference between the total energies of the dissociatively and molecularly adsorbed configurations.

## 3. Results and Discussion

To assess the intrinsic stability of the exposed surfaces, the optimized bulk structures were used to construct slabs thicker than 1 nm. The atomic coordinates were subsequently relaxed at fixed bulk lattice parameters, allowing surface and subsurface reconstruction while retaining the structural constraint imposed by the underlying crystal. The energy difference per structural unit between the slab and the corresponding bulk reference is positive and substantial for every compound considered (Table 1). These values reflect the energetic cost of disrupting metallic or covalent bonds during the bulk-to-surface transition, in contrast to the cleavage of weak interlayer interactions in van der Waals materials. The normalization per structural unit was retained because surface formation in these three-dimensional systems induces relaxation extending beyond the outermost layer into the subsurface.

We first established the hydrogen-adsorption energetics of the pristine surfaces as a reference for their predicted HER response. As in the theoretical works mentioned in the introduction, we considered HER in acidic media, corresponding to the chemisorption of hydrogen on the surface. Calculations were performed for both an isolated hydrogen adsorbate and configurations in which all identified active sites were occupied. For NbAs, TaAs, and NbP, the two chemically inequivalent surface terminations were treated independently. At low hydrogen coverage, VAl_3_, PtGa, and PtAl exhibit hydrogen-adsorption enthalpies in a HER-relevant range, whereas at full occupation, favorable adsorption energetics are also obtained for NbAs and NbP. The sensitivity of the substrate-hydrogen bond to vibrational displacement was estimated by elongating the optimized bond by 0.04 Å and recalculating the total energy. This value is commonly chosen to describe the vibrational properties of chemical bonds because, in subsequent calculations of vibrational frequencies and zero-point energy, it reduces the fraction. The resulting energy changes are typically of the order of 2~3 meV to and ~16 meV (NbP) ~90 meV (PtGa). These contributions do not modify the qualitative ordering of the hydrogen-adsorption energetics or the comparison between pristine and oxidized surfaces.

**Figure 1:**
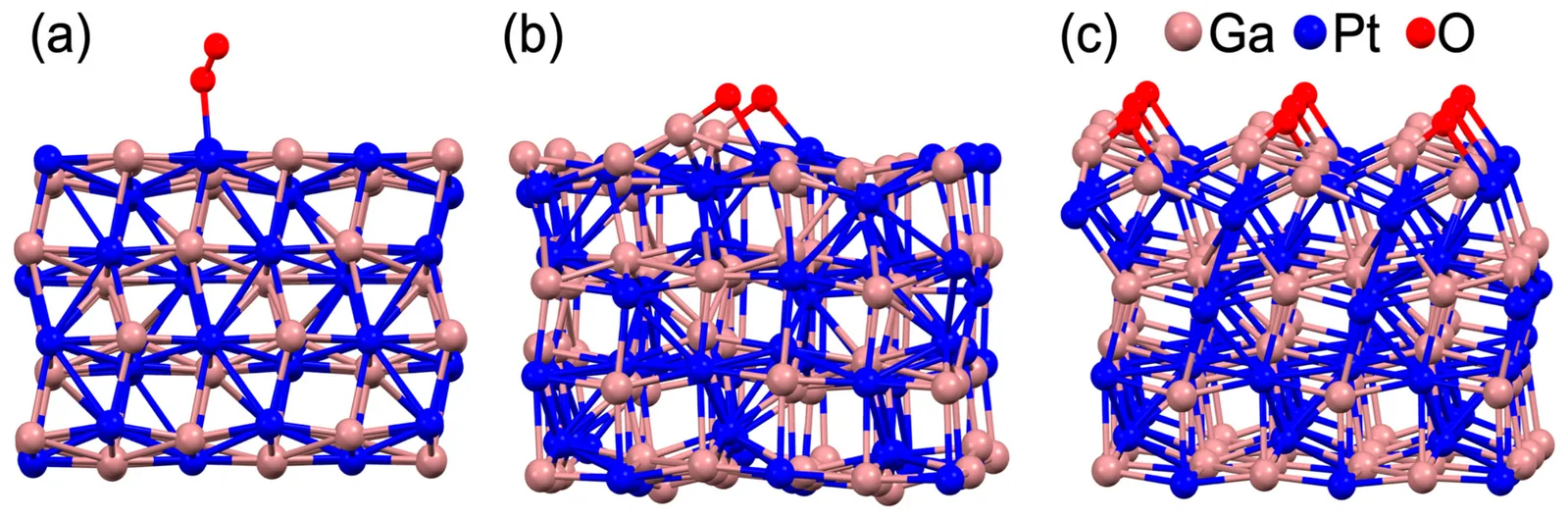
Optimized atomic structures of the PtGa(001) surface with (**a**) molecularly adsorbed O_2_, (**b**) dissociatively chemisorbed oxygen, and (**c**) the highest oxygen coverage considered in this study.

Having established the reference behavior of the pristine surfaces, we next examined their thermodynamic tendency towards oxidation under ambient conditions. Molecular O_2_ physisorption (Figure 1a) ranges from favorable to weakly unfavorable depending on the compound and surface termination. This initial adsorption step, however, does not determine the overall oxidation tendency. Oxygen dissociation (chemisorption, Figure 1b) and the subsequent formation of oxygen-covered surfaces are strongly exothermic throughout the investigated series, with calculated energy gains ranging from approximately 1.7 to 7.5 eV per O_2_ molecule. Thus, even where molecular physisorption is not independently stabilized, the dissociatively adsorbed state remains strongly favored thermodynamically. Under realistic conditions, oxidation of the entire surface (Figure 1c) is therefore expected to nucleate preferentially at vacancies, steps, edges, and other undercoordinated sites and subsequently propagate across the exposed surface.

**Figure 2:**
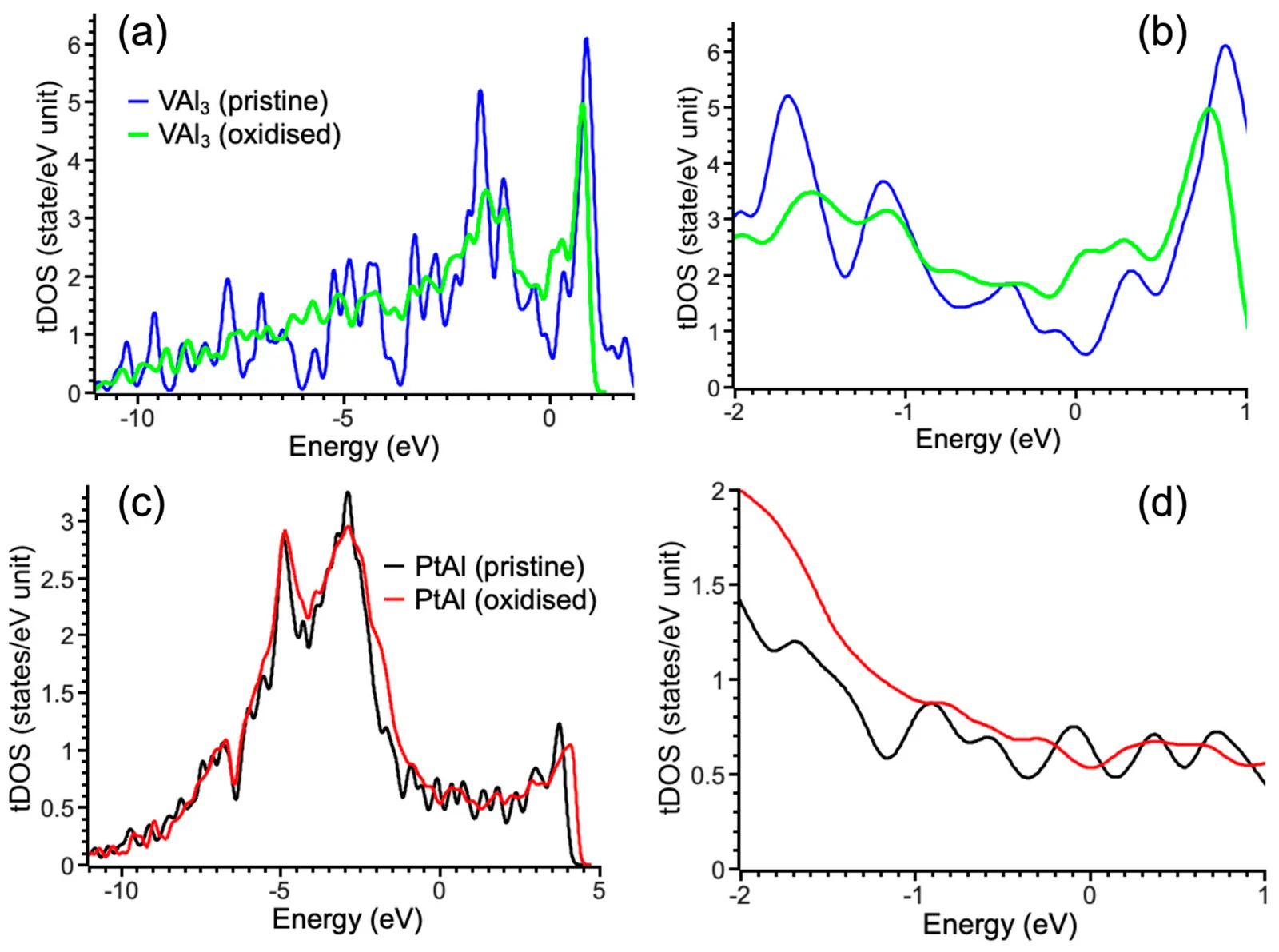
Total electronic densities of states calculated for pristine and oxidized surfaces of VAl_3_ (**a**,**b**) and PtAl (**c**,**d**). Panels (**b**,**d**) show enlarged views of the near-Fermi-level region. The color scale in panels (**a**,**b**) is the same. The color scale in panels (**c**,**d**) is the same. The Fermi energy is set to zero.

Near-Fermi-level electronic states, including topology-derived surface states, have frequently been invoked to explain the catalytic response of topological materials [26]. The calculated density of states shows that oxidation substantially redistributes the electronic spectral weight around the Fermi level, thereby altering the electronic environment of the adsorption sites (Figure 2). The catalytically relevant interface formed after atmospheric exposure consequently cannot be represented as a weakly perturbed version of the pristine topological surface.

For oxidized VAl_3_, adsorption of an isolated hydrogen atom is strongly exothermic, with dH(HER_ox_) = −6.10 eV/H. Such a large magnitude indicates excessive binding in the dilute-coverage limit rather than an intrinsically optimal HER condition. When all identified adsorption sites are occupied, the adsorption enthalpy is reduced to −0.22 eV/H. Including the estimated vibrational contribution gives an adsorption free energy of approximately −0.16 eV, close to the characteristic value reported for Pt, approximately −0.1 eV [27]. The high-coverage configuration therefore provides a more relevant representation of the hydrogen-binding regime than the isolated-adsorbate limit.

A comparable coverage-dependent response is obtained for oxidized PtGa, both terminations of TaAs, the Nb-terminated surface of NbAs, and NbP. In these systems, oxidation markedly modifies the near-Fermi-level electronic structure without eliminating hydrogen-adsorption energetics compatible with HER activity. The convergence of chemically and structurally distinct compounds towards this common behavior demonstrates that oxidation does not simply passivate the topological surface. Instead, it generates a reconstructed catalytic interface whose adsorption properties are still influenced by the electronic structure and surface chemistry of the parent compound. These results align with the observed effects of oxidation of transition metals (see, for a review, the work of B. He et al. [28]), driven by a combination of features in the electronic structure and surface morphology [29].

PtAl constitutes the principal exception. Surface oxidation shifts the hydrogen-adsorption enthalpy from approximately −0.2 eV/H on the pristine surface to values exceeding −3 eV/H at high coverage, indicating excessive hydrogen binding and an unfavorable desorption regime. This change accompanies the loss of the pronounced near-Fermi-level spectral feature in the oxidized-surface density of states (Figure 2d). The discrepancy between the ideal stoichiometric PtAl and its experimentally reported catalytic activity underscores the importance of chemical and structural inhomogeneity in real samples. Recent investigations of Pt_x_Al_y_ compounds have revealed similarities between PtAl and metallic Pt in both their XPS spectra and catalytic behavior [30,31], supporting an interpretation in which Pt segregation or Pt-rich surface regions provide active sites with electronic structures and chemical stabilities closer to those of metallic Pt.

## 4. Conclusions

In conclusion, our results redefine the surface chemistry underlying catalysis in three-dimensional topological materials. The cleavage or preparation of these compounds necessarily disrupts metallic or covalent bonding, generating undercoordinated sites that provide a strong thermodynamic driving force for oxygen dissociation and oxidation of extended surfaces under ambient conditions. Consequently, the pristine surfaces commonly employed in theoretical descriptions are unlikely to represent the catalytic interfaces probed experimentally. Despite substantial reconstruction of the electronic states near the Fermi level, the oxidized surfaces of most investigated compounds retain hydrogen-adsorption energetics compatible with efficient hydrogen evolution, whereas in selected systems, oxidation yields an even more favorable response at high hydrogen coverage. The chemically distinct topological materials considered here therefore converge towards a common behavior in which the parent topological compound acts as an electronically active substrate for an oxidized catalytic surface. PtAl represents an instructive exception, demonstrating that this reconstruction is not universally beneficial and that the resulting activity remains sensitive to the composition and electronic structure of the reconstructed layer. More broadly, these findings resolve an apparent inconsistency in the literature, in which similar catalytic performance has been attributed to markedly different topological band features. They establish that reliable structure–activity relationships in topological catalysis require the explicit treatment of surface oxidation, reconstruction, and catalyst evolution under realistic environmental and reaction conditions, which have been reported in several works but are almost entirely ignored. Topological descriptors derived from pristine surfaces alone are therefore insufficient; the relevant catalytic entity is the dynamically formed interface between the topological parent material, with outstanding electrical properties, and its chemically reconstructed surface.

## Figures and Tables

**Table 1:** Calculated surface-formation, oxygen-adsorption, oxygen-dissociation, and hydrogen-adsorption energetics for pristine and oxidized surfaces. E_surf_ is the surface-formation energy per structural unit; ΔH and ΔG are the enthalpy and Gibbs free-energy change for molecular O_2_ physisorption; E(O_chem_) is the energy change associated with oxygen dissociation; and dH(HER_pure_) and dH(HER_ox_) are the hydrogen-chemisorption enthalpies on pristine and oxidized surfaces, respectively. Values in parentheses correspond to occupation of all identified adsorption sites by hydrogen or, for E(O_chem_), to the highest oxygen coverage considered. The quantities e_vib_ and e_vib_^ox^ denote the changes in total energy produced by elongating the substrate–hydrogen bond by 0.04 Å on pristine and oxidized surfaces, respectively. The chemically inequivalent terminations of NbAs, TaAs, and NbP are reported separately.

System	E_surf_ (eV/unit)	dH(HER_pure_) (eV/H)	e_vib_ (meV)	O_2_ Physisorption		E(O_chem_) (eV/mol)	dH(HER_ox_) (eV/H)	e_vib_^ox^ (meV)
				ΔH (eV/mol)	ΔG (eV/mol)			
VAl_3_	2.01	−0.18 (−0.01)	8.20 (7.99)	−0.16	−0.04	−7.53 (−6.92)	−6.10 (−0.22)	32.4 (30.0)
PtGa	3.87	−0.55 (−0.15)	97.3 (92.2)	0.28	0.39	−5.90 (−3.67)	−1.69 (−0.42)	151.6 (1.2)
PtAl	3.70	−0.19 (−0.19)	7.5 (6.9)	−0.73	−0.61	−4.01 (−4.56)	−4.45 (−3.06)	2.1 (29.8)
NbAs	1.42	Nb-side 5.18 (−0.18)	Nb-side 2.72 (2.87)	Nb-side 3.32	Nb-side 3.44	Nb-side −5.63 (−6.51)	Nb-side −1.75 (−0.14)	Nb-side 107.18 (20.71)
		As-side 6.05 (6.07)	As-side 8.07 (11.73)	As-side 6.20	As-side 6.32	As-side −1.71 (−3.05)	As-side −2.64 (−1.83)	As-side 12.13 (13.30)
TaAs	1.86	Ta-side −0.56 (−1.07)	Ta-side 3.2 (2.4)	Ta-side −3.21	Ta-side −3.09	Ta-side −5.77 (−5.0)	Ta-side 0.94 (−0.37)	Ta-side 2.0 (1.1)
		As-side −1.38 (−1.09)	As-side 6.4 (6.1)	As-side 0.66	As-side 0.78	As-side −3.96 (−3.29)	As-side 0.82 (−0.15)	As-side 1.0 (14.5)
NbP	1.49	Nb-side −0.24 (−0.85)	Nb-side 5.9 (2.0)	Nb-side −2.63	Nb-side −2.51	Nb-side −5.63 (−3.95)	Nb-side −1.03 (−0.39)	Nb-side 34.8 (36.2)
		P-side −0.66 (−0.18)	P-side 16.1 (15.2)	P-side 0.94	P-side 0.12	P-side −4.80 (−4.67)	P-side 1.09 (0.66)	P-side 12.7 (9.27)

## Data Availability

The data supporting this article are available from the corresponding author upon reasonable request.
